# Biological activity validation of a computationally designed Rituximab/CD3 T cell engager targeting CD20+ cancers with multiple mechanisms of action

**DOI:** 10.1093/abt/tbab024

**Published:** 2021-10-22

**Authors:** Wenyan Cai, Jianbo Dong, Sachith Gallolu Kankanamalage, Allison Titong, Jiadong Shi, Zhejun Jia, Bo Wang, Cai Huang, Jing Zhang, Jun Lin, Steven Z Kan, Shuhua Han, Joe Zhou, Yue Liu

**Affiliations:** 1 Ab Studio Inc., Hayward, CA 94545, USA; 2 Ab Therapeutics Inc., Hayward, CA 94545, USA; 3 Genor Biopharma Co. Ltd., Shanghai 201203, P.R.C

**Keywords:** CD20/CD3, therapeutic antibodies, bispecific antibodies, T cell engager antibodies, computer-aided antibody design

## Abstract

**Background:**

Bispecific T cell engaging antibodies (TEAs) with one arm targeting a cancer antigen and another arm binding to CD3 have demonstrated impressive efficacy in multiple clinical studies. However, establishing a safety/efficacy balance remains challenging. For instance, some TEAs have severe safety issues. Additionally, not all patients or all cancer cells of one patient respond equally to TEAs.

**Methods:**

Here, we developed a next-generation bispecific TEA with better safety/efficacy balance and expanded mechanisms of action. Using the computer-aided antibody design strategy, we replaced heavy chain complementarity-determining regions (HCDRs) in one Rituximab arm with HCDRs from a CD3 antibody and generated a novel CD20/CD3 bispecific antibody.

**Results:**

After series of computer-aided sequence optimization, the lead molecule, GB261, showed great safety/efficacy balance both *in vitro* and in animal studies. GB261 exhibited high affinity to CD20 and ultra-low affinity to CD3. It showed comparable T cell activation and reduced cytokine secretion compared with a benchmark antibody (BM). ADCC and CDC caused by GB261 only killed CD20+ cells but not CD3+ cells. It exhibited better RRCL cell killing than the BM in a PBMC-engrafted, therapeutic treatment mouse model and good safety in cynomolgus monkeys.

**Conclusions:**

Thus, GB261 is a promising novel TEA against CD20+ cancers.

**Statement of Significance**

GB261 is a next-generation T cell engager bispecific antibody with enhanced safety, efficacy, and manufacturability balance. In addition, GB261 consists of multiple mechanisms of action, and therefore is good at cancer clearance and countering drug resistance development.

## INTRODUCTION

Immunotherapy that utilizes the body’s immune system against tumors is a promising field of cancer research [1–3]. In recent years, exciting technologies and platforms have been developed for the intricate design and production of anticancer immune reagents such as antibodies and CAR-T cells [4–7]. Among these, bispecific T-cell-engaging antibodies (TEA) have attracted interest [8–11]. BiTE, the most well-known TEA, is a recombinant bispecific protein that has two linked scFvs from two different antibodies: one targeting a cell-surface molecule on T cells (for example, CD3ε), and the other targeting antigens on the surface of malignant cells [10, 12, 13]. BiTEs bind to tumor antigens and T cells simultaneously, and mediate T-cell responses, thus killing tumor cells. While BiTEs have the advantages of relatively simple recombinant production and purification, and a molecular weight enabling tissue penetration, they also have disadvantages of short half-life (2.1 h) and inconvenient drug administration (infusion pump needed) [14]. Recently, scientists from Genentech and Regeneron reported novel bispecific TEA with native human IgG format, which target CD20, a B cell marker, and the CD3 component of the T cell receptor, triggering redirected killing of B cells [15, 16]. For example, it was shown that in cynomolgus monkeys, low doses of REGN1979 (CD20/CD3 bispecific antibody (BsAb)) caused prolonged depletion of B cells in peripheral blood with a serum half-life of approximately 14 days [15]. A phase I clinical trial study suggested that REGN1979 has acceptable safety profile as well as preliminary antitumor activity [17]. While most IgG-like bispecific TEAs show promising efficacy *in vitro* and *in vivo*, some of them have safety concerns to be addressed [18–20]. For instance, REGN1979 caused two deaths in clinical studies because it elicited cytokine release syndrome (CRS) [20]. Other than safety concerns, another limitation for almost all current IgG-like bispecific TEAs is lack of Fc effector functions: antibody-dependent cellular cytotoxicity (ADCC) and complement-dependent cytotoxicity (CDC), which are the major functions of Rituximab, a widely used anti CD20+ tumor monoclonal antibody [21, 22]. This may limit the benefits of these BsAbs to certain patients who respond to ADCC and/or CDC. To resolve these problems and develop the next generation of bispecific TEAs with better safety and more mechanisms of action for targeting cancer heterogeneity and diverse cancer drug resistance, we designed a novel CD20/CD3 BsAb, GB261, with ultra-low CD3 binding affinity, which allows efficient T cell activation only in the presence of CD20+ target cells but not in the presence of CD20- cells. Because this novel BsAb retains ADCC and CDC functions that targets CD20+ cancer cells only, but not CD3+ T cells *in vitro*, it has potential to trigger cancer cell killing faster and more efficiently in pathophysiological settings where the T/B cell ratios are extremely low. The reason for this effect is that even before encountering T cells, it could kill cancer cells via ADCC/CDC immediately after being injected. This initial cancer cell killing event may enhance the T cell/cancer cell ratio and the chance for BsAb to engage T cells. Once the BsAb binds to both cancer cells and T cells, it activates T cells and further induces T cells-mediated cancer cell killing. In addition, the enhanced ratio of natural killer (NK) cells and macrophages to cancer cells would further increase the ADCC/CDC/antibody-dependent cellular phagocytosis (ADCP) functions, thereby further exacerbating the cancer cell killing. Because of this novel mechanism, GB261 can be a better therapeutic candidate compared with other CD20/CD3 BsAbs.

To design GB261, we employed the computer-aided antibody design (CAAD) strategy. With this, we designed multiple common VLs and selected the VL of Rituximab as the lead common VL. We replaced the heavy chain complementarity-determining region (HCDR) of one Rituximab arm with CD3 HCDRs, modified these parental VH sequences to differentiate their chemico-physical characteristics, reduced the immunogenicity of the BsAb, increased the stability of the BsAb via backmutations, humanized the VH regions, and introduced mutations in the Fc domain to enhance heterodimer formation. The detailed design process and the mutations used in the generation of GB261 are published elsewhere [23, 24]. The CAAD was preferred over the traditional antibody discovery methods because CAAD significantly decreases the timeframe of antibody development. CAAD can be used to generate structural modifications easily and more successfully in the therapeutic antibodies because it can predict the functionality of those modifications. Moreover, when multiple modifications are possible in a given structure, CAAD can help select the ones with best safety, efficacy, and developability balances. Therefore, the CAAD approach would potentially lead to next generation of therapeutic BsAbs with improved clinical benefits.

## MATERIALS AND METHODS

### Strategy for designing GB261

**Figure 1:**
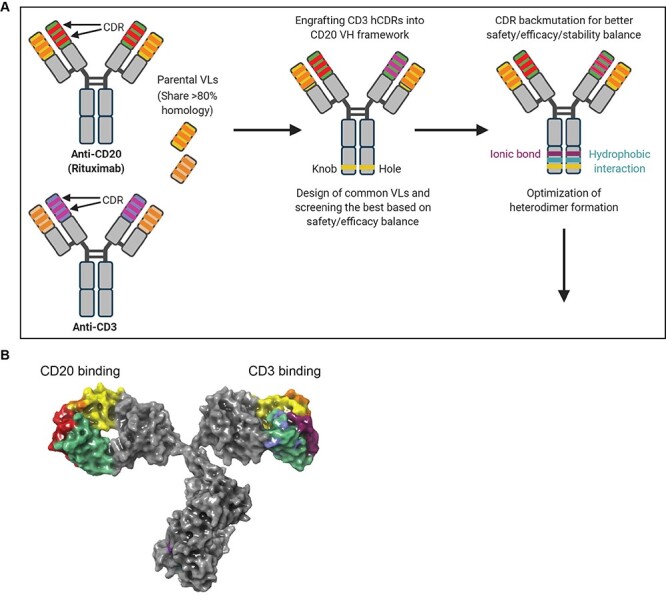
**Computer-aided design process of the “imbalanced” BsAb GB261.** A) HCDRs in one arm of the parental CD20 (Rituximab analog) was replaced by the HCDRs of parental CD3 antibody to construct the preliminary CD20/CD3 BsAb. Several common VL sequences that share homology to VL (CD20) and VL (CD3) including Rituximab VL that may lead to loss of binding to CD3 but maintains binding to CD20 were designed and paired with the two VHs. These IgG-like BsAbs with different common VL candidates were constructed in the “knob into hole (KIH)” format comprising of mutations T366Y and Y407T (R). Then, T cell activation assays were performed to test the BsAb molecules and the version with Rituximab VL was selected as the lead BsAb due to its high safety/efficacy balance. Then, new mutations S354Y (Hydrophobic interaction) and Q347E (Ionic interaction) in the Fc domain were introduced to further improve heterodimer formation (ETYY format) [24]. In addition, the pI of the two BsAb VHs was differentiated by introducing new mutations in the VH non-CDR framework regions. The deimmunization mutations were also introduced. B) The final lead molecule, GB261, was selected based on great safety/efficacy balance as well as great manufacturability represented by high expression level, high heterodimer formation percentage, easy purification process, and designed with great biochemical and biophysical characterization features. The 3D structural prediction of GB261 is shown with its CD20 binding arm, CD3 binding arm, and the Fc domain. This structure was generated with Schrodinger BioLuminate software. (Schrödinger Release 2020–3: BioLuminate, Schrödinger, LLC, New York, NY, 2020. https://www.schrodinger.com/products/bioluminate).

GB261 was designed using BioLuminate software (Schrödinger Release 2020-3: BioLuminate, Schrödinger, LLC, New York, NY, 2020. https://www.schrodinger.com/products/bioluminate.) [25–27] and the design process is explained in detail in Figure 1.

### Cell lines and PBMC

Raji (Human B-lymphoma cell line, CCL-86; Lot No:63905419) and Jurkat (Human T cell lymphoblast-like cell line, Clone E6–1, TIB-152) were from ATCC. Raji cell line stably expressing EGFP and Luciferase (Raji-GFP-Luciferase) was supplied by Biocytogen. They were cultured in RPMI 1640 (Thermo fisher) medium supplemented with 10% fetal bovine serum (FBS) (GIBCO) at 37 °C, 5% CO_2_. Human peripheral blood mononuclear cells (hPBMC) were purchased from AllCells (Alameda, CA) and HumanCells Bio (San Jose, CA). Rituximab resistant Raji cells (RRCL) were developed as described in Supplementary Materials.

### Bispecific antibody purification

GB261 was produced by co-transfecting plasmids encoding the CD20 heavy chain, CD3 heavy chain and their common light chain into Expi293 cells and purified using a Protein A column on an AKTA Explorer 100 purification system and further purified using cation exchange chromatography. Detailed method is described in Supplementary materials.

### Bispecific antibody cell binding assays

Binding of BsAb to CD3 and CD20 expressing cells was determined by flow cytometry. Briefly, 2 × 10^5^ Jurkat (CD3+/CD20−) or Raji (CD20+/CD3−) cells were incubated for 30 min at 4 °C with serial dilutions of BsAb or hIgG1 isotype control antibodies. Then, cells were washed with DPBS (GIBCO) containing 1% Bovine Serum Albumin (BSA) (Proliant Biologicals), and incubated with Cy3-conjugated Goat anti-human IgG (Jackson ImmunoResearch) at 1:1000 dilution for 30 min at room temperature (RT). The cells were washed twice, and their geometric mean fluorescence intensity (MFI) was measured using fluorescence-activated cell sorting (FACS).

### *In vitro* cancer cell killing, T cell activation, and total T cell number assessment assays

Raji-GFP-Luc or RRCL-GFP-Luc cell were mixed with PBMC from healthy donors at ratios as indicated, plated in 96-well flat bottom plates (2.0 × 10^5^ cell/well), treated with antibodies, and cultured in a CO_2_ incubator for 48 h. The cells were washed twice with DPBS containing 2% FBS, stained with CD69-PE (BD Biosciences) and CD2-APC (BD Biosciences) antibodies (1:100) for 45 min at RT, then washed twice again and analyzed using FACS. Cancer cell killing was determined by the GFP positive cell percentage, T cell activation was determined by the CD69+/CD2+ cell percentage, and total T cell number was determined by the CD2+ cell percentage.

### Cytokine release assays

The cell culture supernatants from the previous *in vitro* tumor cell killing, T cell activation, and total T cell number assessment assays were used for cytokine release assays. The cytokines IFN-γ, IL-2, and TNF-α were assessed using ELISA MAX Deluxe kits (BioLegend) according to manufacturer’s instructions.

### ADCC assays

Target cells were washed once with PBS, incubated with calcein AM (7.5 μM) at 37 °C for 30 min in dark, washed three times with PBS, resuspended in RPMI1640 with 10% FBS (2 × 10^5^ cells/ml) and then added to a 96-well plate (50 μL/well). Diluted antibodies (100 μL) were added to each well. The plate was incubated at RT for 60 min, and then PBMC (effector cells) in 50 μL of media were added to each well (5 × 10^4^ cells/well). The plate was incubated at 37 °C for 4 h and centrifuged at 1000 rpm for 10 min. Supernatants were transferred into a 96-well black wall plate, and fluorescence was read at 520 nm (emission) with 485 nm excitation.

### CDC assays

Target cells were labeled with calcein AM as described in ADCC assays. The labeled cells were plated in 96-well plates (1 × 10^5^ cells/well in 50 μL), and antibodies at different dilutions were added (100 μL/well). The plate was incubated at RT for 15 min, and 10% complement-enriched human serum was added to the wells (50 μL/well). The plate was incubated at RT for 45 min, the cells were washed three times with PBS containing 0.1% BSA and stained with 7-Aminoactinomycin D (7AAD) (2 μL/well) at RT for 15 min in dark. The cells were analyzed by flow cytometry. The percentage of dead cells was defined as the calcein+/7AAD+ cell fraction.

### Antibody-mediated cell bridging assay

Detailed method is described in Supplementary materials.

### Animal studies

All animal studies were performed in compliance with the ARRIVE guidelines.

#### Immediate treatment and therapeutic treatment mouse models

All animal handling, care, and treatment procedures were performed in accordance with the guidelines approved by the Institutional Animal Care and Use Committee (IACUC) of Biocytogen Boston (following the guidelines by the Association for Assessment and Accreditation of Laboratory Animal Care (AAALAC)). Eight- and nine-week-old female B-NDG mice (strain NOD.Cg-*Prkdc^scid^ Il2rg^tm1Bcgen^*/Bcgen) (Biocytogen) were used for the immediate treatment and therapeutic treatment studies, respectively. The investigators were blinded, and only the sponsors were aware of the group allocation during each step of the experimental process.

In the immediate treatment study, the mice were randomized into three groups, with four mice per group, based on bodyweights. RRCL-GFP-Luc cells (5 × 10^5^ cells/mouse) were injected with PBS into mice in the control group through i.v. administration. RRCL-GFP-Luc cells (5 × 10^5^ cells/mouse) were mixed with hPBMC (5 × 10^5^ cells/mouse) and either BM or GB261 and injected via the lateral tail vein at a dose of 1.5 mg/kg at day 0. Two additional doses of antibodies were injected to mice on day 7 and day 14 as well. On day 20, the study was terminated when the mice in the control group reached humane endpoints.

In the therapeutic treatment study, RRCL-GFP-Luc cells were injected into mice (1 × 10^5^ cells/mouse) through i.v. administration. After 3 days, the mice were divided into three groups at five mice/group. At day 4, the HLA-DR3 positive hPBMC (HumanCells Bio) were mixed with either PBS (control), BM, or GB261, and administered intravenously. Each mouse in the treatment groups received 5 × 10^5^ hPBMC, and 20 μg of antibodies at day 0. At day 7, an additional dose of antibodies was administered to mice. On day 14, the study was terminated when the mice in the control group reached humane endpoints.

In both studies, the mice were imaged twice weekly by bioluminescence imaging (BLI), and the BLI was quantitated by the software Living Image 4.7 (Perkin Elmer). Bodyweights of mice were also measured twice a week. The mice were euthanized by CO_2_ inhalation followed by a secondary physical method (such as cervical dislocation). The data were analyzed and calculated using MS Excel and GraphPad Prism 8. In the immediate and therapeutic treatment studies, the treatment and control groups were compared using unpaired T test and Kruskal–Wallis test with Dunn’s multiple comparison test, respectively. A p-value of <0.05 was considered statistically significant.

#### Tumor relapse mouse model

All animal handling, care, and treatment procedures stated in this study were performed in accordance with the standard at Biocytogen Beijing. B-NDG mice were used for the study. Only the sponsors were aware of the group allocation and the investigators were blinded during each step of the experimental process. The mice were randomized by bodyweight into three groups, with four mice per group. Each mouse was intravenously injected with 5 × 10^5^ Raji-Luc cells, 2.5 × 10^6^ hPBMC, and 60 μg of antibodies (PBS for the control group). Raji-Luc cells were cultured to logarithmic phase and the hPBMC were thawed and incubated overnight before using for the experiments. The tumor load in mice was imaged three times weekly by BLI using IVIS Lumina LT (Perkin Elmer). Bodyweights of mice were also measured three times a week. The mice were monitored for 49 days and the study was terminated. At the study end or when the mice reached humane endpoints, they were euthanized with CO_2_. The treatment and control groups were compared using unpaired T test and a p-value of <0.05 was considered statistically significant.

#### Cynomolgus monkey study

Before starting the experiment, the animals were raised in the experimental site for at least 1 week. During this period and the experimental process, the health of the animals was monitored by the veterinarian. Healthy cynomolgus monkeys (2.5–4.5 kg，3–6 years old) were randomly divided into two groups: 0.5 mg/kg (Group 1) and 5 mg/kg (Group 2). The investigators were not blinded and were aware of the group allocation during each step of the experimental process. Each group had two animals, including one male and one female. GB261 was administered by single-dose intravenous infusion. Animal breeding, quarantine, drug delivery and blood collection were conducted by Laboratory of Guangxi Guidong Primate Development and Experiment Co., Ltd. (Guangxi, China) in accordance with relevant guidelines and regulations under IACUC protocol No.GD20200604 approved by the Animal Management and Use Committee of Guangxi Guidong Primate Development and Experiment Co., Ltd. The serum concentrations of GB261 and cytokines as well as percentages of T cells and B cells in blood were determined at different time points.

### Data analysis

The data were analyzed by Prism (GraphPad). IC_50_ and EC_50_ values were determined using Four-parameter nonlinear regression analysis.

## RESULTS

### Designing process of GB261

This means that the two parental antibodies used to construct the bispecific antibody are 1) Rituximab, and 2) A CD3 antibody that has a VL with > 80% homology to that of Rituximab [23]. To develop a novel CD20/CD3 BsAb with great safety/efficacy balance as well as great manufacturability, the following features were introduced in the design process: 1) HCDR from the parental CD3 antibody were grafted in one arm of the Rituximab analog heavy chain; 2) Rituximab light chain was maintained as the common light chain; 3) CD3 binding affinity was significantly reduced to increase safety; 4) ADCC/CDC effector functions specific for cancer cells were maintained to broaden the mechanisms of action; 5) biochemical and biophysical features of the two Fv regions of the IgG-like BsAb arms were differentiated to facilitate better isolation of the BsAb heterodimer; 6) backmutations (CD3 HCDRs) and other modifications were introduced in the heavy chains to enhance the stability, deimmunization, and humanization; 7) new Fc mutations were introduced to improve heterodimer production and purification [24] (Fig. 1). After designing and screening, safety, efficacy and manufacturability of the lead molecule were characterized.

### GB261 has an ultra-low CD3 binding

**Figure 2:**
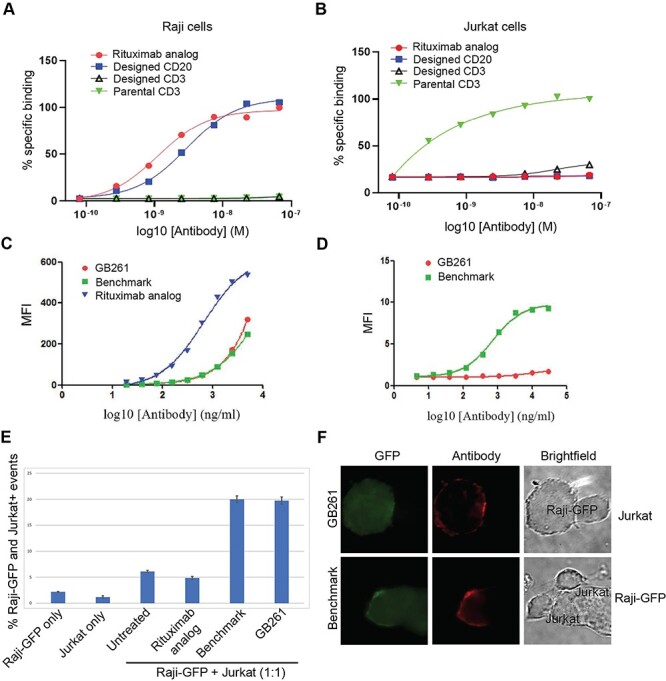
**GB261 is weaker than BM in CD3 binding but is comparable in CD20 binding.** A) Binding of designed CD20 (Rituximab analog with common VL) and CD3 antibodies (CD3 homodimer with common VL) to CD20+ Raji cells, compared with those of the parental CD20 (Rituximab analog) and CD3 antibodies. Cells were incubated with antibodies, labeled with Cy3-conjugated goat anti-human antibody, followed by FACS. The binding was presented as the percentage of cells positive for staining. B) Antibodies tested in Figure 2A were examined for binding to CD3+ Jurkat cells using the same method. C) Binding of GB261, BM and Rituximab analog to Raji cells. The cells were labeled with a Cy3-conjugated goat anti-human antibody and analyzed using FACS. The binding was quantified as the mean fluorescent intensity (MFI) of staining. D) Binding of GB261 and BM to Jurkat cells. E) Raji-GFP cells were mixed with Jurkat cells pre-stained with CellVue Claret Far Red fluorescent dye at 1:1 ratio and incubated overnight with 20 μg/ml antibodies, followed by the detection of cells by FACS. The events double positive for GFP and fluorescent dye were counted as bridged Raji-GFP and Jurkat cells. F) Raji-GFP cells were mixed with unstained Jurkat cells at 1:1 ratio, incubated with 20 μg/ml antibodies, and stained with DyLight 594-conjugated goat anti-human IgG. Two representative images showing the bridging of Raji-GFP cells and Jurkat cells in the presence of antibodies are shown. The display of the images has been adjusted non-uniformly for representation.

To ensure that antibodies we designed have a good safety/efficacy balance, the binding of our redesigned Rituximab analog and CD3 homodimer antibody with the common VL to CD20+ Raji cells and CD3+ Jurkat cells were compared with that of parental antibodies. Our redesigned CD3 antibody had a significantly lower binding to CD3+ Jurkat cells than the parental CD3 homodimer antibody, whereas our redesigned Rituximab analog and the parental Rituximab analog had similar binding to CD20+ Raji cells (Fig. 2A, B). Neither the redesigned nor the parental CD3 antibodies had any detectable binding to Raji cells. Similarly, neither the redesigned nor the parental Rituximab analog significantly bound to Jurkat cells. Based on these studies and manufacturability data, we designed the final lead molecule, named as GB261, with significantly decreased binding to CD3 compared with the BM (Fig. S1).

The binding of GB261 to CD3+ Jurkat cells was much weaker than that of BM (Fig. 2D). However, the binding of GB261 to CD20+ Raji cells was similar to that of BM, whereas the binding of the Rituximab analog to CD20+ cells was much stronger that those of GB261 and BM (Fig. 2C). Moreover, both GB261 and the BM mediated bridging between Raji and Jurkat cells (Fig. 2E, F), suggesting a mechanism by which the antibodies mediate cancer cell killing.

### GB261 has balanced safety/efficacy *in vitro*

**Figure 3:**
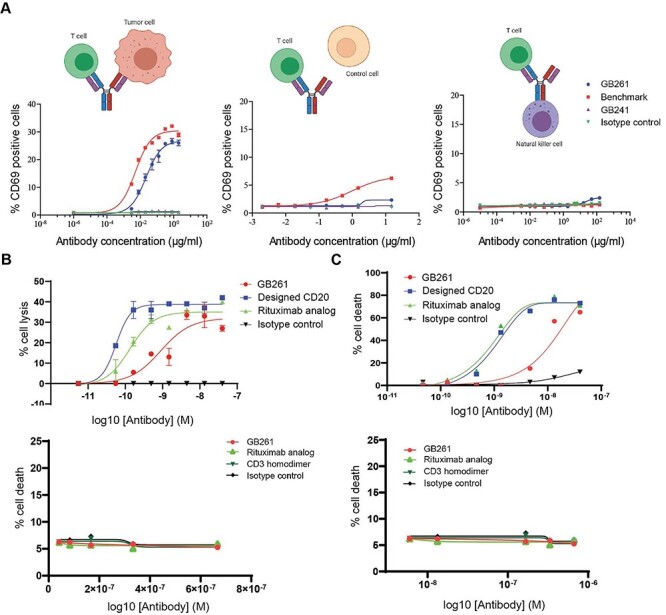
**GB261 has balanced safety/efficacy.** A) Jurkat (T cell) activation by GB261, BM, GB241, and an isotype IgG as performed by co-incubating with Raji (left), CHO cells (middle) and NK92-CD16 cells (right) at 1:1 ratio. The cells were labeled with anti-CD69 antibodies conjugated to PE and analyzed by flow cytometry. B) Upper panel, ADCC induced by either GB261, Designed CD20, Rituximab analog, or an isotype IgG. Raji cells labeled with Calcein AM were treated with antibodies and incubated with NK-92-CD16 cells. Lower panel examines whether the GB261-induced ADCC kills T cells that it binds to. Human PBMC were incubated with GB261, Rituximab analog, a CD3 mAb, and the isotype control antibody. T cell viability was analyzed by FACS. C) Upper panel, CDC induced by either GB261, Designed CD20, Rituximab analog, or an isotype IgG. Raji cells labeled with Calcein AM were treated with antibodies and incubated with complement-enriched human serum. Lower panel, Jurkat cells were incubated with antibodies as described in C Upper panel and the viability of the cells were analyzed by FACS.

To compare the effects of GB261 and BM on T cell activation, Jurkat cells were co-cultured with Raji (CD20+) cells at a ratio of 1:1, treated with different concentrations of antibodies, and then stained with anti-human CD69-PE antibody and analyzed using FACS. In the presence of Raji cells, both GB261 and BM induced a dose-dependent effect on the Jurkat cell activation, but GB261 was less efficient than the BM in Jurkat cell activation (Fig. 3A, left). GB241, an analog of Rituximab, and an isotype control antibody, which has similar Fc segment to GB261 but does not have a relevant Fab segment, did not induce Jurkat cell activation. Moreover, GB261 did not activate Jurkat cells in the presence of CD20- cells, whereas BM activated Jurkat cells at high concentrations due to its stronger binding to CD3 (Fig. 3A, middle). In addition, GB261 did not induce Jurkat cell activation in the presence of NK92-CD16 cells, suggesting that GB261 cannot induce T cell activation through Fc/FcR interaction (Fig. 3A, right). These data suggest that GB261 may have better safety than the BM.

To compare the Fc effector functions of GB261 and the Rituximab analog, ADCC and CDC assays were performed. At low concentrations, GB261 was less effective than the Rituximab analog and Rituximab homodimer derived from GB261 with a common VL (Designed CD20) in eliciting ADCC on CD20+ Raji cells, whereas at high concentrations, GB261 exhibited similar effect to the Rituximab analog and Designed CD20 (Fig. 3B, upper panel). Similar results were observed in CDC assays (Fig. 3C, upper panel). These antibodies did not induce ADCC or CDC on CD20- Jurkat cells (Fig. 3B, C, lower). In other words, GB261 only induced ADCC and CDC on CD20+ cells, but not on CD3+ T cells, suggesting its good safety/efficacy balance.

**Figure 4:**
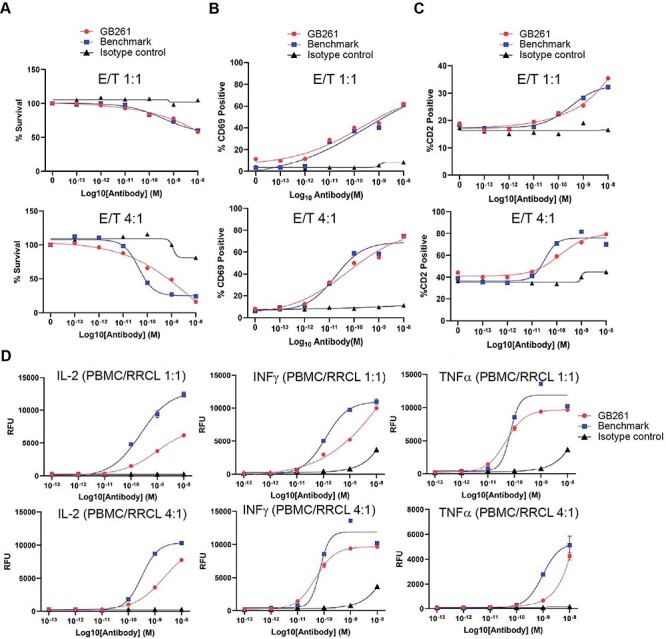
**GB261 and BM has similar effects on tumor cell killing, T cell activation and total T cell number, but GB261 induces less cytokine release compared with BM.** hPBMC were mixed with RRCL-GFP-Luc cells at E:T 1:1 or 4:1 ratios and incubated with test antibodies GB261 and BM, or an isotype control antibody for 48 h. The cells were labeled with CD2-APC and CD69-PE antibodies followed by analyzing with FACS. A) RRCL-GFP-Luc killing was assessed by the percentage of survived GFP positive cells. B) T cell activation was determined by the percentage of CD2+/CD69+ cells, and C) total T cell number was assessed by the percentage of CD2+ cells. D) The experiment was performed as described above, and the release of cytokines was detected by ELISA using the cell culture supernatants.

To compare the effects of GB261 and BM on cancer cell killing, T cell activation and total T cell number in Rituximab-resistant cells which generally decreases the CD20 antigen density by ~ 3 folds compared with Raji cells [28, 29], RRCL-GFP-Luc cells were incubated with hPBMCs either at 1:1 ratio or 1:4 ratio in the presences of different concentrations of testing antibodies. At 48 h post-incubation, GFP+ cell surviving percentage, T cell activation, and total T cell number were detected, respectively. As shown in Figure 4, GB261 and BM had similar effects on RRCL killing (A), T cell activation (B) and total T cell number (C), showing the efficacy of GB261.

Immunotherapy can cause CRS when immune cells are activated and release large amounts of cytokines into the body [30, 31]. High levels of cytokines can result in increased inflammation. Thus, they can be harmful and cause organ failure and even death [32]. Therefore, a successful immunotherapeutic antibody should be able to induce T cell activation and cancer cell killing but not elicit a strong CRS. To this end, we compared the effects of GB261 and BM on the release of several cytokines. GB261 induced less IL-2, IFNγ and TNFα secretion than BM, suggesting that GB261 has less potential to induce CRS (Fig. 4D).

### GB261 retains favorable efficacy at low T/B cell ratios *in vitro* and *in vivo*

Previous studies have shown that when cancer B cells overgrow in patients, the B/T cell ratio could significantly increase [33]. Therefore, we analyzed whether GB261 with Fc effector functions is better than the Fc-silenced BM in low T/B cell ratios due to initial cancer cell elimination and T/B ratio re-adjustment by ADCC and/or CDC. To do so, we performed *in vitro* and *in vivo* studies comparing cancer killing by GB261 and BM at different T/B ratios. GB261 was more efficacious than BM when PBMC/RRCL and PBMC/Raji cell ratios were low *in vitro* (Fig. S2).

**Figure 5:**
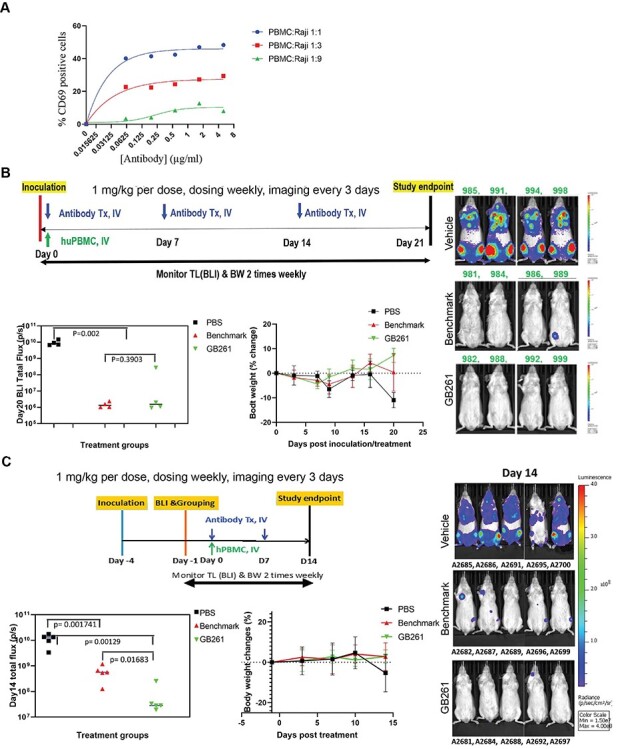
**GB261 has high efficacy at low T/B cell ratios.** A) hPBMC and Raji-GFP-Luc cells were mixed at ratios of 1:1, 1:3 and 1:9 and incubated with indicated concentrations of GB261 or an isotype IgG. T cell activation was assessed by measuring CD69+/CD2+ T cells by FACS. The background T cell activation by the isotype control antibody was subtracted from the values obtained for GB261. B) RRCL-GFP-Luc cells were mixed with HLA matching (HLA-DR3+) hPBMC at 1:1 ratio. Then, the cells were mixed with PBS, GB261, or BM, and subsequently injected into mice by i.v. (four mice/group). Each mouse received 5 × 10^5^ RRCL-GFP-Luc cells and 5 × 10^5^ PBMC, with or without the antibodies. An amount of 20 μg of antibodies were administered at day 0 and day 7, and 60 μg of antibodies were administered at day 14. The mice were monitored for a total period of 21 days and the tumor luminescence was imaged every 3 days. The workflow, total tumor luminescence on day 20, and the percentage body weight change of the mice from the initial weight are shown in the upper, lower left, and lower right panels, respectively. This study was performed by Biocytogen Boston Corp as a contract research service. C) The mice were first inoculated by RRCL-GFP-Luc cells. After 4 days, the HLA matching hPBMC were mixed with PBS (Control) or 1 mg/kg test antibodies GB261 or BM, and injected into mice by i.v (five mice/group). As a result, the T:B cell ratio is about 1:8. Each mouse received 5 × 10^5^ RRCL-GFP-Luc cells and 5 × 10^5^ PBMC. The antibodies were administered at day 0 and day 7 and the mice were monitored for a total period of 14 days. The tumor luminescence was imaged every 3 days. The workflow, total tumor luminescence on day 14, and the percentage body weight change of the mice from the initial weight are shown in the upper, lower left, and lower right panels, respectively. This study was performed by Biocytogen Boston Corp as a contract research service.

To compare this effect *in vivo*, we used a PBMC-engrafted mouse model which can be administered with the lymphoma cells. While the PBMC-engrafted mice generally have the limitation of inducing graft-versus-host disease (GVHD) as opposed to the humanized mice [34], we utilized PBMC and lymphoma cells with matching HLA to overcome this issue. First, RRCL-GFP-Luc cells were injected into the mice and after 3 days, they were randomly divided into three groups. At day 4, together with PBMC, the first group was administered with PBS, second group with BM, and the third group with GB261. Although both BM and GB261 induced RRCL killing in PBMC-engrafted mice, GB261 was much more effective than BM, and both antibodies induced comparable body weight change in mice (Fig. 5C). However, in another study, when RRCL cells were injected with PBMC and antibodies at the same time (lower luciferase signal implies higher effector/target cell ratio at the beginning of the study), GB261 and BM had similar effects on tumor size decrease and body weight in mice (Fig. 5B). The significant increase in the number of RRCL cells when they are injected 4 days before antibody treatment, as shown by the day 3 (post-antibody treatment) luminescence signal increase compared with when RRCL and antibodies are injected together (data not shown), decreases the T cell/RRCL ratio, thus reducing BM efficacy, whereas GB261 can increase the T cell/RRCL ratio via ADCC/CDC, consequently retaining its efficacy. This is likely due to the increased T cell activation when the T cell/cancer cell ratios are higher, as demonstrated by our *in vitro* data (Fig. 5A).

**Figure 6:**
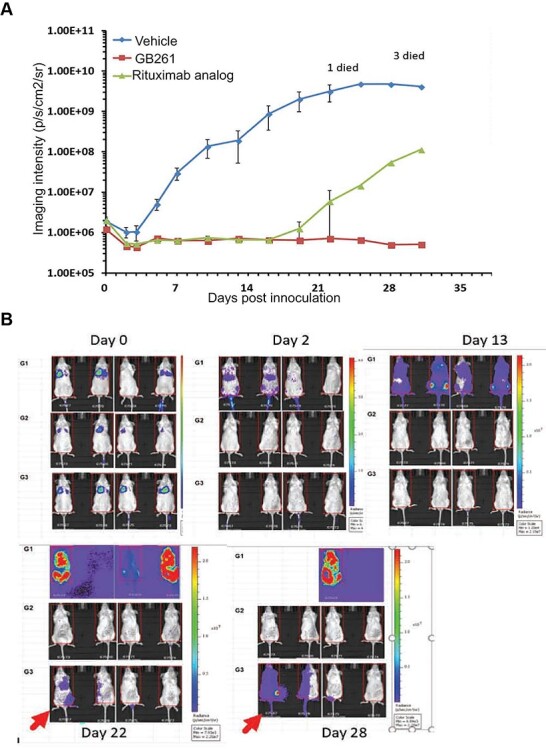
**GB261 prevents tumor relapse in an *in vivo* mouse model.** Raji-GFP-Luc cells and hPBMC were mixed with either PBS, GB261, or Rituximab analog, and injected into mice by i.v. A total of four animals were used per each treatment group. The mice received 5 × 10^5^ Raji-GFP-Luc cells, 2.5 × 10^6^ PBMC, and 60 μg of each antibody. They were imaged for the tumor luminescence at 15 min post-i.v., and then at day 2, day 3 and every 3 days after that. The mice were monitored for a total period of 31 days. The tumor volume was quantified (A) and the luminescent images of the mice are shown (B). This study was performed by Beijing Biocytogen Co., Ltd. as a contract research service.

To compare the efficacies of GB261 and the Rituximab analog in lymphoma cell killing, Raji-GFP-Luc were treated with PBS, GB261, and Rituximab analog, respectively, mixed with PBMCs, and then injected into the mice. Raji cell proliferation was monitored using luciferase imaging. GB261 effectively mediated Raji cell killing through the whole experimental period. Rituximab analog also effectively mediated Raji cell killing initially for 16 days, but the tumors were able to relapse following that initial period (Fig. 6). These data suggest that GB261 can maintain the lymphoma cell killing efficacy longer than Rituximab, likely due to its multiple mechanisms of action.

### GB261 shows safety in cynomolgus monkeys

**Figure 7:**
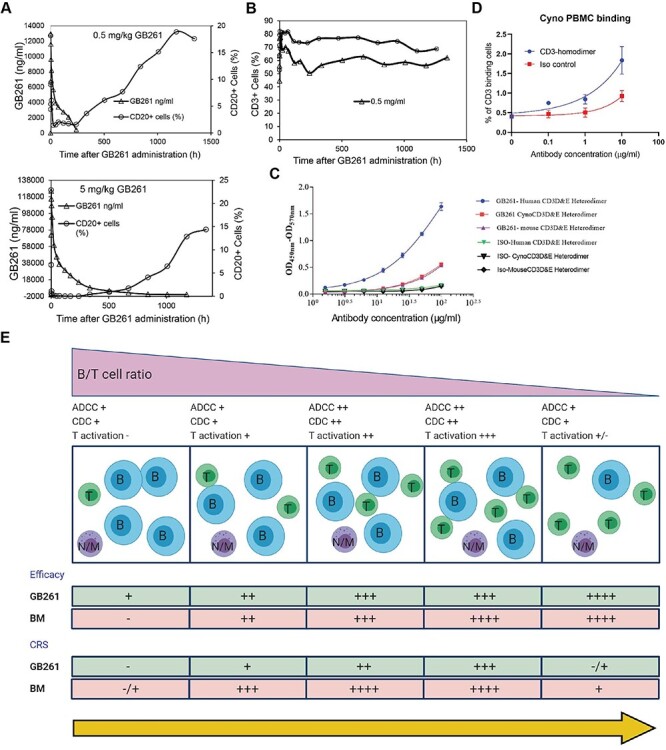
**Effects of GB261 on CD20+ and CD3+ lymphocytes in Cynomolgus monkeys.** A) CD20+ lymphocytes and GB261 levels in Cynomolgus monkey blood after administration of 0.5 mg/ml or 5 mg/ml of GB261. Lymphocytes are mean of two Cynomolgus monkeys. GB261 levels are mean of nine Cynomolgus monkeys. B) CD3+ lymphocytes of Cynomolgus monkeys after administration of 0.5 mg/ml or 5 mg/ml of GB261. Data are mean of two Cynomolgus monkeys. C) The binding of GB261 or an isotype control antibody at indicated concentrations to recombinant human, Cynomolgus, and mouse CD3D and E heterodimers were assessed by ELISA. The binding was measured as the optical density (OD) values at 450 nm and 570 nm. D) Binding of CD3 homodimer and an isotype control antibody to Cynomolgus PBMC, determined by FACS. E) A model depicting the mechanisms of action of GB261, compared with those of BM.

Next, we determined whether GB261 treatment depletes circulating B cells and whether it causes any unexpected toxicity in cynomolgus monkeys. The animals were treated with either 0.5 or 5 mg/kg of GB261, and levels of circulating B cells, T cells and inflammatory cytokines were monitored for 8 weeks. After infused with different doses of GB261, CD20+ cells (B cells) were depleted rapidly, and CD20+ could not be detected after 24 h. With the extension of monitoring time, CD20+ recovered to a certain extent (Fig. 7A). The CD3+ T cells increased to a certain extent and remained higher than the levels before administration after 56 days (Fig. 7B). In addition, there was no significant amount of cytokines detected except for IL-6. This is probably caused by the significantly lower binding of GB261 to cynomolgus CD3 compared with its binding to human CD3 (Fig. 2D, 7C and 7D). Together with our *in vitro* data, these data suggest that GB261 also exhibits great safety/efficacy balance in primates.

### GB261 has favorable manufacturability features

GB261 has a good expression in CHO cells (~6 g/L). The heterodimer formation percentage is high which enables its high-efficient production (Fig. S3A). In addition, the product has a good purity (Fig. S3B, C), aggregation resistance, and thermostability (Fig. S3D).

## DISCUSSION

While the discovery of therapeutic antibody candidates is a relatively straightforward process, their development to clinically approved drugs often confront with unexpected problems. In this study, we present that GB261, a novel CD20/CD3 BsAb designed from Rituximab, retains Fc effector function that only targets B cells and gains T cell engager function with reduced CRS. Currently, there are number of CD20/CD3 TEAs under various stages in clinical development such as mosunetuzumab [35] (Genentech), plamotamab [36] (Xencor), REGN1979 [15] (Regeneron), Epcoritamab [37] (Genmab), and glofitamab [35] (Genentech). Most of these antibodies have been designed to have the 1–1 antibody format, whereas glofitamab has a 2–1 format which may increase the potency of the antibody [38]. Similar to these antibodies, GB261 which contains the 1–1 antibody format also contains the T cell activation capability. As T cell activation is a widely studied strong cancer cell killing mechanism, GB261 should have similar cancer cell killing efficacy as compared with other TEAs in clinical development. However, the focus of developing GB261 is to combat the cancer heterogeneity, drug-resistance development, and antibody-dependent CRS development which cannot be fully addressed by improving antibody efficacy. For example, antibodies with both 1–1 and 2–1 formats, but with limited mechanisms of action may not completely clear cancer and would allow residual cancer to survive. This may later develop into drug-resistant cancer. In addition, CRS is a major problem frequently associated with TEAs [30, 31]. Previous studies have shown that cytokine release is dependent on the binding affinity of the CD3 binding arm [39, 40]. Based on their design, the current TEAs in clinical development do not have lowered CD3 binding. Here, SPR data show that the CD3 arm of GB261 displays reduction by several folds compared with a benchmark antibody, potentially addressing the safety issue of CRS development. It is likely that this difference is similar between GB261 and the other CD20/CD3 benchmark TCEs in clinical development.

Due to its retained Fc effector functions and resultant multiple mechanisms of action, GB261 can counter the drug resistance of cancer cells better than agents with limited mechanisms of action. Cancers are comprised of heterogenous cell populations in terms of both therapeutic response (e.g. cells with diverse mutations) and resistance development. GB261 has the potential to clear the residual cancer cell populations that might survive the treatment with a drug with a single MOA, thereby enhancing cancer clearance. In addition, GB261 can counter different modes of drug resistance development in cancer cells due to its multiple MOA compared with a single MOA agent. These drug resistance mechanisms include but not limited to PD-L1/PD-1 overexpression to reduce T cell activation [41], CD46/CD55/CD59 overexpression to inhibit CDC [42] and CD16A ***V*** & ***F*** genotypes to affect ADCC [43]. This decreases the chance of cancer relapse in patients. In addition, the patients who have already developed drug-resistant tumors by the above-mentioned resistance mechanisms would benefit from the treatment by GB261 due to its multiple MOA. This is exemplified by our *in vivo* studies that show a single dose of GB261 could prevent the cancer relapse for the entire duration of the study, whereas a single dose of Rituximab analog which contains a single mechanism of action could only prevent the relapse up to 16 days although it decreased the cancer burden initially, likely due to the expansion of survived lymphoma cells following antibody clearance (Fig. 6).

In addition, GB261 is engineered to have ultra-low CD3 binding affinity in order to minimize CRS. Therefore, GB261 has the potential to overcome safety issues caused by extra T cell activation. We show that compared with the benchmark CD20/CD3 TEA, GB261 has a low CD3 binding affinity (Fig. 2), which enables GB261 to activate T cells in the presence of CD20+ target tumor cells, but not in their absence. Also, the ultra-low CD3 binding of GB261 minimizes the ADCC/CDC-mediated T cell killing caused by the Fc/FcR interactions but retains ADCC/CDC-mediated B cell killing.

Although GB261 elicits less cytokine release compared with BM, it does not compromise its efficacy. In fact, it retains T cell activation and cancer killing capacity on RRCL cells comparable to that of BM. Furthermore, the cancer cell killing capacity of GB261 is comparable to that of BM at higher T/B cell ratios, but is higher than that of BM at lower T/B cell ratios that mimic the pathophysiological conditions. This high efficacy of GB261 at low T/B cell ratios can also be attributed to its multiple mechanisms of action. GB261 can target cancer cells via Fc effector functions ADCC/CDC/ADCP, or T cell-mediated killing. At initial stage of treatment when the B cancer cells are more prevalent than T cells, GB261 can eliminate cancer cells via Fc effector functions with the aid of NK cells, macrophages (M), and the complement pathway. This would reduce the cancer cell numbers and result in higher T/B cell, NK/B cell, and M/B cell ratios. Therefore, at later stages of the treatment, the T cell-mediated B cell killing would be more prominent because this action is more efficient at higher T/B cell ratios where the T cells are activated more efficiently. The increased NK/B and M/B cell ratios would further enhance the Fc-mediated elimination of cancer cells at later stages of the treatment, assisting the action of T cell-mediated B cancer cell killing (Fig. 7E). This is further supported by the observation that GB261 inhibited cancer cell growth at low doses, in a dosing study *in vivo* (Fig. S4). For antibodies without Fc effector functions, additional agents with Fc effector functions might initially be required to increase the T/B cell ratio. This is the reason that REGN1979 is more efficient when Rituximab is used to increase the T/B cell ratio before REGN1979 administration [44]. Later on, when most cancer B cells are killed, there are more free T cells which could still be activated by BM but not GB261 (Fig. 1). This assertation is supported by both our *in vitro* data and the mouse models, where GB261 shows superior cancer cell killing than BM in a PBMC-engrafted mouse model if RRCL cells were injected first, followed by PBMC cells and then antibodies were co-injected 4 days later when the T/B cell ratio decreased in animals. In contrast, GB261 exhibits similar cancer killing effect to BM when RRCL and PBMC cells were co-injected with antibodies when the T/B cell ratio is relatively high.

GB261 exhibits a favorable manufacturability. It has good expression, and a high heterodimer/homodimer formation ratio. Also, the product is easily purified with good yield and purity. In addition, the purified antibody has a good thermostability and is aggregation resistant. The results suggest that GB261 would meet the Chemistry Manufacturing and Controls (CMC) requirements for successful drug development. Taken together, GB261 has a good balance among efficacy, safety, and developability, and has the potential to replace Rituximab as a first-line drug in lymphoma therapy.

## DATA AVAILABILITY

For original data, please contact yue.liu@antibodystudio.com.

## AUTHOR CONTRIBUTIONS

W.C. performed cell-based assays, designed some animal studies, analyzed data and contributed to drafting of the manuscript. J.D. designed antibody engineering work, performed the experiments, and analyzed data. S.G.K. designed some cell-based assays, performed experiments, analyzed data and prepared manuscript. A.T. designed some cell-based assays and analyzed data. B.W. developed the purification strategy of bispecific antibody. J.S. designed some cell-based assays, performed experiments, and analyzed data. Z.J. performed molecular cloning work. C.H. analyzed data and prepared manuscript. J.Z. designed the cyno pre-tox study, J.L., S.Z.K. led the CMC process and produced GB261, S.H. reviewed data from Genor Biopharma, and J.Z. led all research at Genor Biopharma, analyzed the data and revised the manuscript. Y.L. conceived the idea, designed GB261 molecule, analyzed the data and prepared manuscript.

## SUPPLEMENTARY DATA

Supplementary data are available at *ABT* Online.

## Supplementary Material

100821_Supplementary_materials_by_Cai_et_al_tbab024
