# Supplementary material for: Biological activity validation of a computationally designed Rituximab/CD3 T cell engager targeting CD20+ cancers with multiple mechanisms of action
Source: Antib Ther. 2021 Oct 22;4(4):228–41. doi: 10.1093/abt/tbab024 (PMC8597964; doi:10.1093/abt/tbab024)
Supplement: 100821_Supplementary_materials_by_Cai_et_al_tbab024 [file 100821_supplementary_materials_by_cai_et_al_tbab024.docx]

**Biological activity validation of a computationally designed Rituximab/CD3 T cell engager targeting CD20+ cancers with multiple mechanisms of action**

*Wenyan Cai^1^, *Jianbo Dong^1^, Sachith Gallolu Kankanamalage^1^, Allison Titong^1^, Jiadong Shi^1^, Zhejun Jia^1^, Bo Wang^1^, Cai Huang^2^, Jing Zhang^3^, Jun Lin^3^, Steven Z. Kan^3^, Shuhua Han^3^, Joe Zhou^3^, and Yue Liu^1,2^

^1^ Ab Studio Inc., 3541 Investment Blvd., Suite 3, Hayward, CA 94545

^2^ Ab Therapeutics Inc., a JHBP company, 3541 Investment Blvd., Suite 2, Hayward, CA 94545

^3^ Genor Biopharma Co. Ltd., a JHBP company, 1690 Zhangheng Road, Building 3, Pudong New District, Shanghai, P.R.C.

*These authors contributed equally to this work

To whom correspondence should be address:

Yue Liu, Ph.D.

Ab Studio Inc.,

3541 Investment Blvd., Suite 3

Hayward, CA 94545

[yue.liu@antibodystudio.com](mailto:yue.liu@antibodystudio.com)

**Statement of Significance:** GB261 is a next generation T cell engager bispecific antibody with enhanced safety, efficacy, and manufacturability balance. In addition, GB261 consists of multiple mechanisms of action, and therefore is good at cancer clearance and countering drug resistance development.

**Keywords:** CD20/CD3, Therapeutic antibodies, Bispecific antibodies, T cell engager antibodies, Computer-aided antibody design

**Supplementary methods**

***Bispecific antibody purification***

GB261 was produced by co-transfecting plasmids encoding the CD20 heavy chain, CD3 heavy chain and their common light chain into Expi293 cells using ExpiFectamine 293 Transfection Kit (Thermo Fisher) according to manufacturer’s instructions. At 72 h after transfection, the cell culture supernatant was centrifuged at 3000 g for 10 min. The supernatant was filtered with a 0.45 µm membrane, and BsAb concentration was measured using a Protein A probe on gator (Probe Life). The BsAb was purified using a Protein A column on an AKTA Explorer 100 purification system (buffer A: PBS, pH=7.4; buffer B: 0.1 M glycine, pH=2.5), dialyzed in PBS (pH=7.4) twice, and then further purified using cation exchange chromatography (POROS GoPure HS Pre-packed Column, Thermo Fisher), with a salt gradient (Buffer A: 20 mM phosphate buffer, pH=7.4; Buffer B: 20 mM phosphate buffer, 1 M NaCl, pH=7.4). The purified BsAb was dialyzed in PBS twice, filtered with a 0.22 µM filter and tested by a T cell activation assay. Then, the Endotoxin content was quantified using the Pierce LAL Chromogenic Endotoxin Quantitation Kit and removed using the Pierce high capacity endotoxin removal columns, according to manufacturer’s protocols.  The BsAb was then filtered again with a 0.22 µm filter before using for the experiments.

***Rituximab resistant Raji cells***

Rituximab resistant Raji cells (RRCL) were developed as follows: Raji or Raji-GFP-Luc cells were treated with 102 µg/ml Rituximab and 10% pooled normal human serum (Innovative Research, Inc., IRLA-SER-23970); Initially, the antibody-containing old media was replaced with fresh antibody-containing media every 3 days up to 2 weeks to allow the cells to become healthy. Then, the serum concentration was increased, and the rituximab concentration was maintained at 102 µg/ml in the media. After 52 days, RRCLs were maintained in media with 10% serum.

***Antibody-mediated cell bridging assay***

Jurkat cells were labelled with the CellVue Claret Far Red dye and washed as described previously ^23^. Raji-GFP cells only or Raji-GFP cells mixed with labeled Jurkat cells at 1:1 ratio in RPMI-1640 containing 10% FCS, treated with antibodies (20 µg/ml), and incubated overnight at 37 °C, 5% CO2. The cells were washed once with FACS buffer and analyzed using FACS. For microscopic assays, the cells were fixed with 4% paraformaldehyde and washed with PBS. Then, the cells were blocked with 10% normal goat serum for 30 min. The cells were stained with DyLight 594-conjugated goat anti-human IgG Fc cross-absorbed antibody for 1 h at RT. The cells were mounted on glass slides, covered with coverslips, and imaged with an Olympus microscope.

***Surface plasmon resonance analysis of antibody binding***

The Surface plasmon resonance (SPR) detection of antibody binding was performed using a Biacore T200 (GE Healthcare) instrument with a CM5 chip, equipped with the Biacore T200 Control Software. The samples were loaded to instrument after centrifuging at 10000 rpm for 3 minutes before the affinity detection. An anti-His antibody at 50 μg/ml was used as the capture antibody and immobilized on CM5. Then, recombinant human CD3D & CD3E heterodimer protein (Sino Biological, Cat.# CT038-H2508H) was fixed by the capture antibody. Then, GB261 and Benchmark were diluted to 800, 400, 200, 100, and 50 nM and added to interact with fixed CD3. Following the program run, the binding-dissociation curves of antibodies in various concentrations were analyzed by the Biacore Evaluation software.

**Supplementary figure legends**

**Figure S1. The binding-dissociation curves of GB261 and Benchmark with human CD3.** The binding-dissociation curves were obtained by analyzing the interaction of human CD3 with GB261 and Benchmark antibodies by Biacore Evaluation software. The CD3 binding interaction was fitted according to the Steady State Affinity binding model. The binding rate constant (ka) and dissociation rate constant (kd) were calculated to obtain the affinity constant (KD). KD=kd/ka (M). The KD values for GB261 and Benchmark are 2.00x10^-06^ M (Chi² (RU²) - 0.00622) and 1.12x10^-09^ M (Chi² (RU²) - 1.6), respectively.

**Figure S2. GB261- and Benchmark-induced cancer cell killing at different E:T ratios.** The RRCL-GFP-Luc cells or Raji-GFP-Luc cells were mixed with human PBMC at E:T ratios of 1:1 (A), 1:3 (B), and 1:9 (C) and incubated overnight at 37 °C with the indicated concentrations of antibodies in the presence of 10% human complement serum. Then, the percentage of cell death relative to the isotype control was determined by FACS based on the GFP+ live cells.

**Figure S3. GB261 has high manufacturability with favorable physico-chemical characteristics.** A) GB261 was purified by Protein A via AEX. B) The GB261 sample was prepared in a reducing and non-reducing labeling buffer and analyzed by capillary electrophoresis (CE). B) Reducing CE-SDS assay of the AEX-purified GB261 is shown here. C) Non-reducing CE-SDS assay of the AEX-purified GB261. D) thermostability (DSF, SLS) and aggregation potential of the AEX-purified GB261.

**Figure S4. In-vivo dosing study of GB261.** The pre-mixed RRCL-GFP-Luc cells and hPBMC at ~1:1 ratio was mixed with either PBS (Control) or different concentrations (per mouse) of GB261 as indicated, and injected into mice by i.v. A total of 3 mice were used per each group. Each mouse received 5 x 10^5^ of Raji-GFP-Luc cells and PBMC. They were imaged for the tumor luminescence at days 3, 7, 10, 14, 17, and 21 post i.v. The tumor volumes were quantified (upper panel) and the luminescent images of the mice are shown (lower panel). This study was performed by Biocytogen Boston Corp as a contract research service.

**Supplementary figures**

**Figure 1**

**
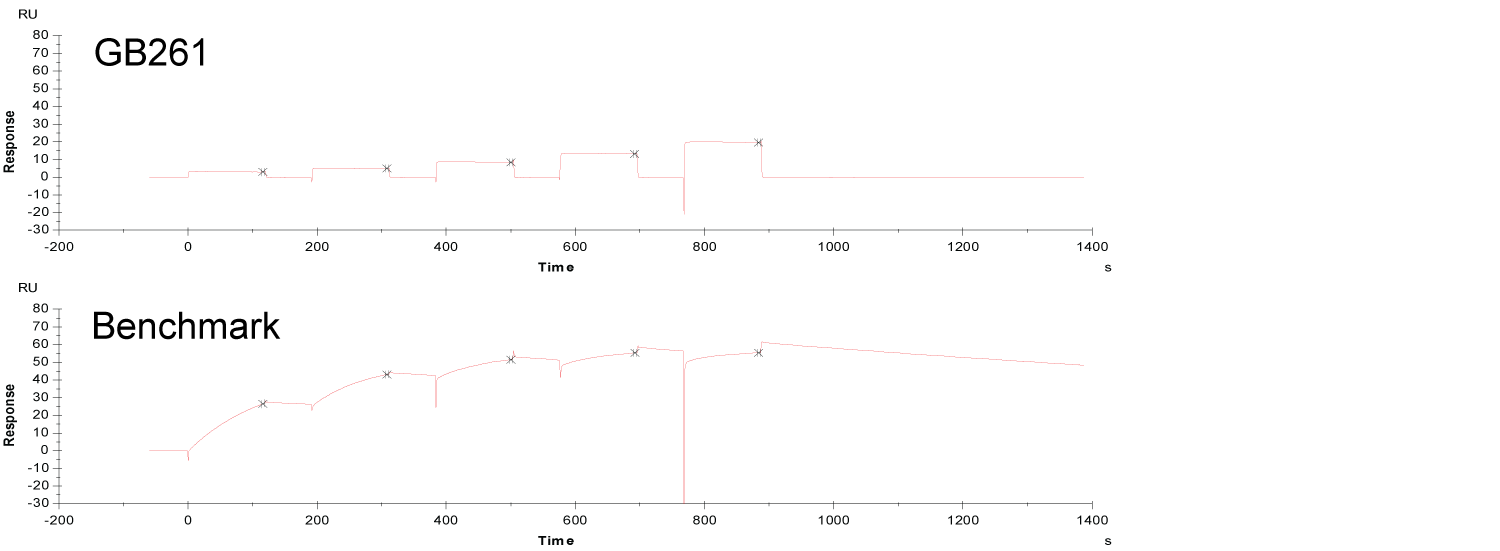
**

**Figure 2**

**
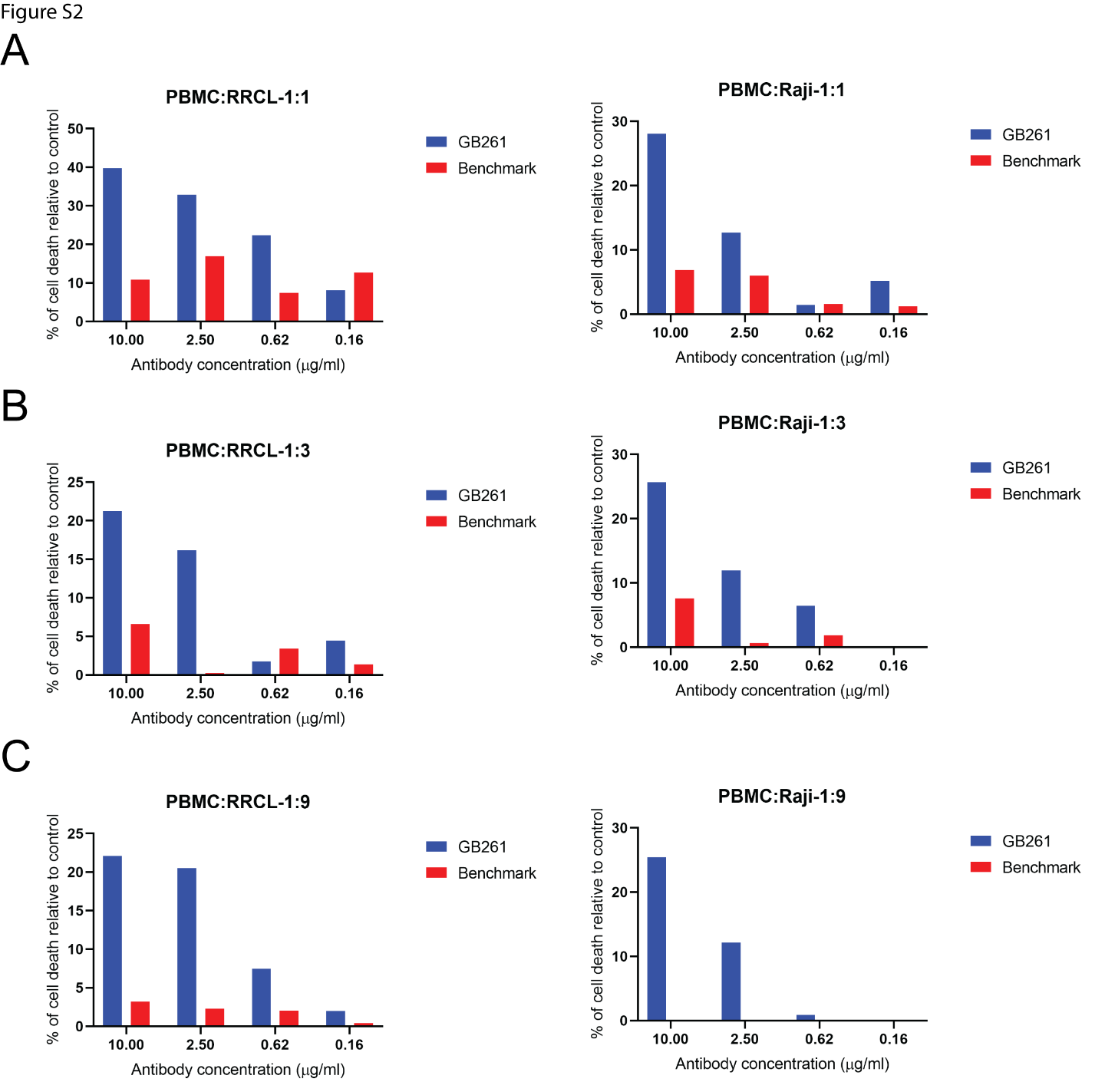
**

**Figure 3**


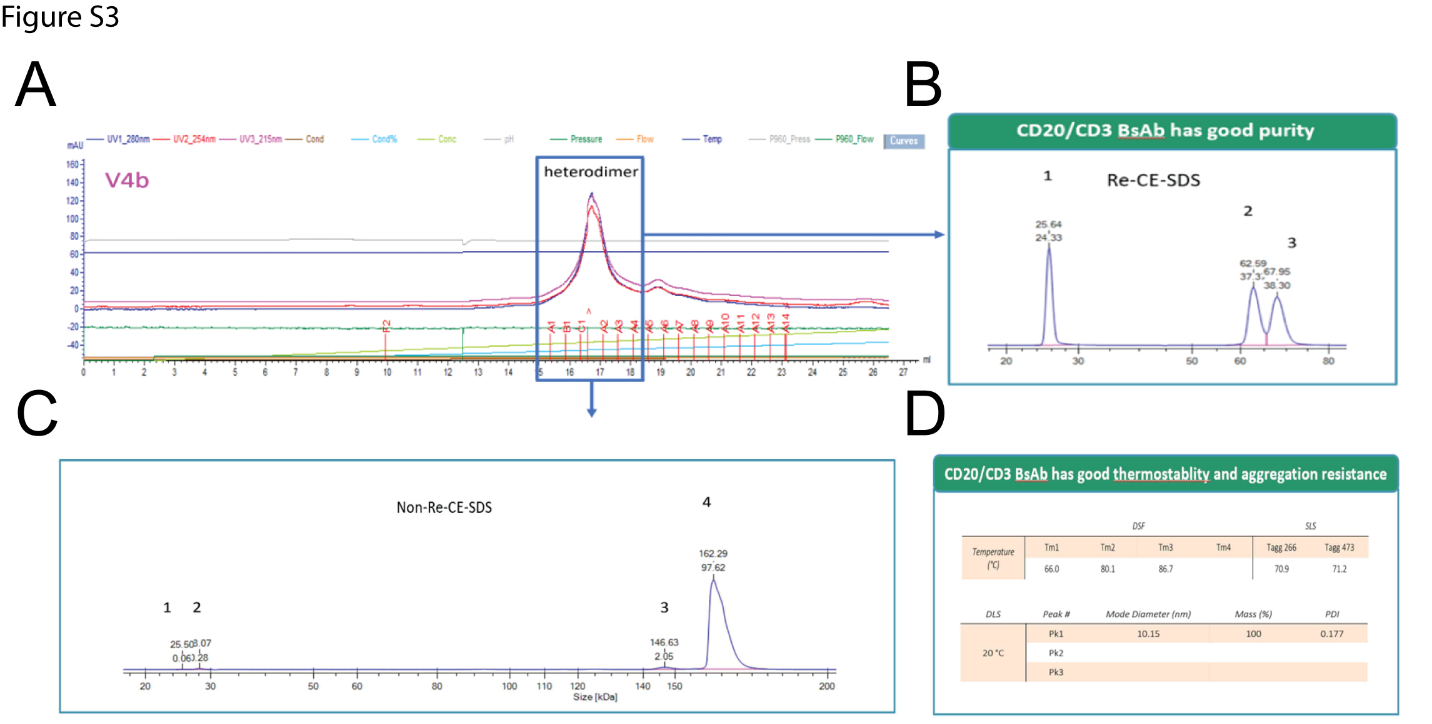


**Figure 4**

**
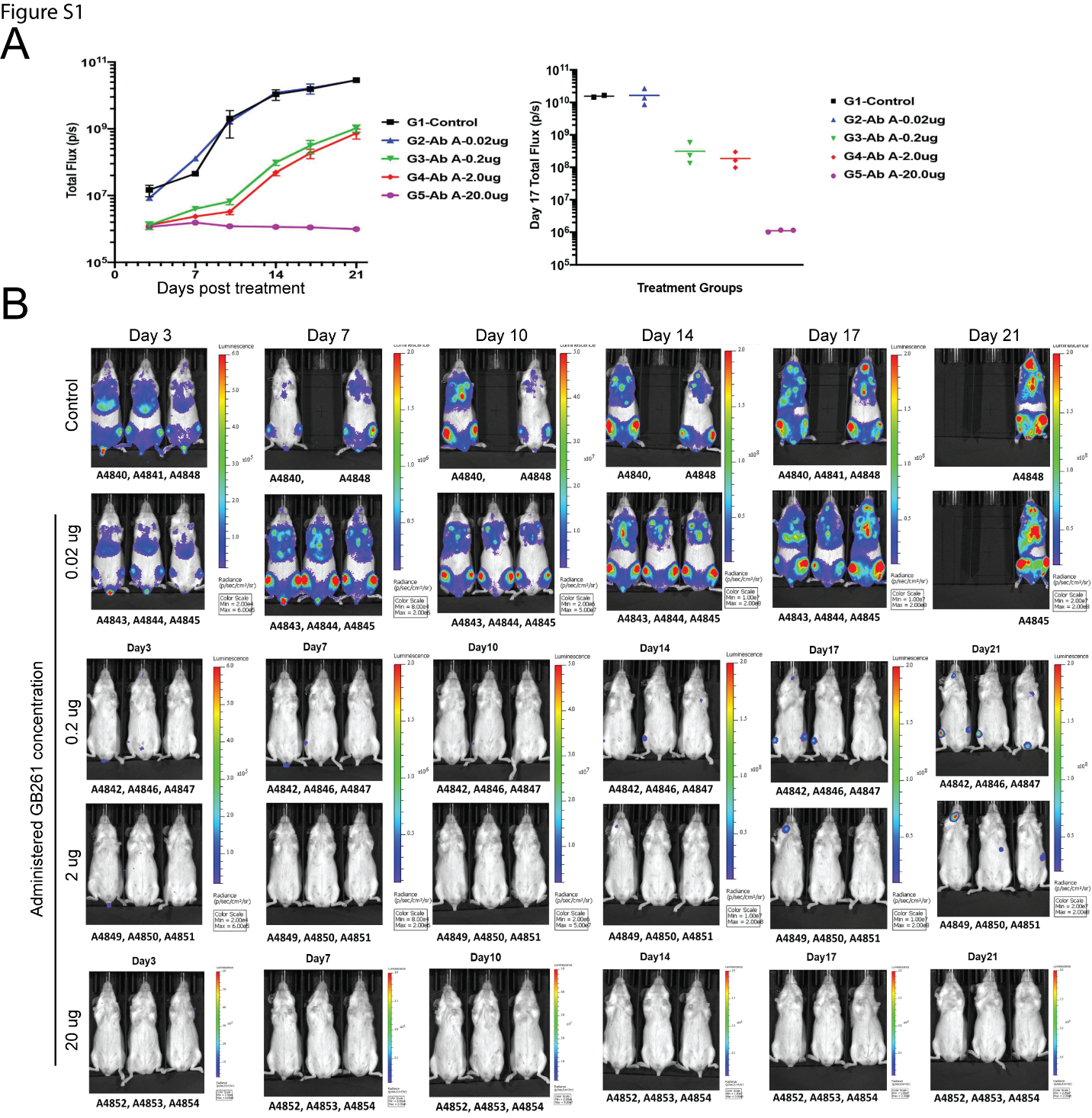
**
